# From Brew to Bronchoscopy: A Case of Hyponatremia That Was Anything but Basic

**DOI:** 10.7759/cureus.81749

**Published:** 2025-04-05

**Authors:** Ghazwan Bahro, Elise J Landa, Dallas J Petroff, Srihita Patibandla, Ali Z Ansari, Natasa Petreska

**Affiliations:** 1 Department of Internal Medicine, Central Michigan University College of Medicine, Saginaw, USA; 2 Department of Pulmonology, MyMichigan Health, Saginaw, USA; 3 Department of Ophthalmology, Idaho College of Osteopathic Medicine, Boise, USA; 4 Department of Internal Medicine, Trinity Health Grand Rapids, Grand Rapids, USA; 5 Department of Pathology and Laboratory Medicine, William Carey University College of Osteopathic Medicine, Hattiesburg, USA

**Keywords:** alcohol-related dehydration, beer potomania, bronchoscopy, chronic obstructive pulmonary disease (copd), fine-needle aspiration (fna), hyponatremia, mediastinal lymphadenopathy, small cell lung cancer (sclc), smoking, syndrome of inappropriate antidiuretic hormone secretion (siadh)

## Abstract

We present the case of a 53-year-old female with a history of chronic alcohol use, hypertension, chronic obstructive pulmonary disease (COPD), and significant smoking history, who presented with severe hyponatremia. Initial laboratory studies revealed a sodium level of 115 mEq/L. The patient reported chronic vomiting, poor oral intake, and heavy alcohol consumption. Her hyponatremia was initially attributed to multiple factors, including alcohol-related dehydration, beer potomania, and potential medication-induced syndrome of inappropriate antidiuretic hormone (SIADH) secretion. However, further evaluation with chest computed tomography (CT) revealed extensive mediastinal lymphadenopathy. Bronchoscopy with fine-needle aspiration confirmed a diagnosis of small cell lung cancer (SCLC). This case highlights the importance of a comprehensive evaluation for hyponatremia, particularly in patients with malignancy risk factors, to avoid overlooking severe underlying conditions such as cancer.

## Introduction

Hyponatremia, characterized by a serum sodium concentration of less than 135 mEq/L, is the most prevalent electrolyte disorder in both inpatient and outpatient settings. It has a 15%-30% prevalence among hospitalized patients [1]. Clinically, hyponatremia is often asymptomatic but can present with a broad spectrum of symptoms, ranging from mild fatigue and nausea to life-threatening complications such as cerebral edema, seizures, coma, and death, particularly when sodium levels fall below 120 mEq/L [2]. The severity of symptoms is influenced by both the degree of sodium deficit and the rapidity of its onset. In acute hyponatremia, where serum sodium levels decline over less than 48 hours, patients face an increased risk of severe neurological complications due to osmotic imbalances that lead to cellular swelling, especially within the brain. In contrast, chronic hyponatremia allows for gradual cerebral adaptation, potentially delaying severe symptoms but requiring even more careful management to prevent complications from overly rapid sodium correction [3].

The pathophysiology of hyponatremia is complex and multifactorial, with numerous potential etiologies classified based on the patient’s volume status: hypovolemic, euvolemic, and hypervolemic hyponatremia. Hypovolemic hyponatremia results from fluid loss (e.g., gastrointestinal losses, diuretic-induced renal losses), while euvolemic hyponatremia is commonly associated with conditions such as the syndrome of inappropriate antidiuretic hormone (SIADH) secretion, which can also contribute to hypervolemic hyponatremia in some instances [4,5]. Hypervolemic hyponatremia is linked to fluid overload states, including heart failure, cirrhosis, and nephrotic syndrome [6]. Accurately identifying the underlying etiology is crucial, as management strategies vary depending on the cause. For hypovolemic hyponatremia, volume repletion remains the cornerstone of treatment, whereas euvolemic and hypervolemic forms often require fluid restriction, sodium repletion, and, in some cases, pharmacologic intervention [4].

Small cell lung cancer (SCLC) is an aggressive neuroendocrine tumor strongly associated with smoking and characterized by rapid growth and early metastasis. It frequently causes paraneoplastic syndromes through ectopic hormone production, including SIADH, which can present as hyponatremia [7]. The diagnosis of SCLC is often challenging due to its nonspecific early symptoms, such as weight loss, cough, and dyspnea. Given its aggressive nature and the significant morbidity associated with paraneoplastic effects, early recognition of SCLC is essential, particularly in patients with high-risk factors such as chronic alcohol use and a substantial smoking history.

## Case presentation

A 53-year-old Caucasian female with a significant past medical history of hypertension, chronic obstructive pulmonary disease (COPD), alcohol use disorder, bipolar disorder, and a 40 pack-year smoking history presented to her primary care physician with complaints of persistent weakness, nausea, poor appetite, and recurrent vomiting over the past two weeks. She described progressive fatigue that limited her daily activities and reported an unintentional weight loss of several pounds during this period. The patient admitted to consuming five to six beers daily while drinking less than 1 liter of water per day. She also acknowledged poor adherence to her prescribed medications, including hydrochlorothiazide for hypertension and olanzapine for bipolar disorder.

At her primary care visit, initial laboratory testing revealed severe hyponatremia, with a serum sodium level of 115 mEq/L. Concerned about the potential for serious complications, her physician referred her to the emergency department for further evaluation and management. Upon arrival, the patient appeared clinically dehydrated but was alert, oriented, and asymptomatic, with no confusion, seizures, or signs of alcohol withdrawal. She reported her last alcohol intake two days before admission and denied experiencing withdrawal symptoms, such as tremors, hallucinations, or seizures.

On physical examination, the patient exhibited dry mucous membranes, poor skin turgor, and low-normal blood pressure despite her history of hypertension. Heart and lung examinations were unremarkable, with no evidence of jugular venous distention or peripheral edema. Neurological assessment revealed no focal neurological deficits, with no signs of encephalopathy or altered mental status. Further laboratory evaluation confirmed severe hyponatremia. Key laboratory results included a serum osmolality of 233 mOsm/kg, urine osmolality of 219 mOsm/kg, and urine sodium levels below 20 mEq/L. Table 1 provides a comprehensive summary of the laboratory findings associated with the patient's condition. Thyroid-stimulating hormone and cortisol levels were within normal limits. The low urine sodium and urine osmolality were more indicative of extra-renal causes of sodium loss, such as alcohol-related dehydration, vomiting, and beer potomania, rather than directly confirming the patient's volume status. Additionally, the use of olanzapine raised concerns for possible SIADH, contributing to the multifactorial nature of her hyponatremia.

**Table 1:** Laboratory findings indicate severe hyponatremia with low serum and urine osmolality.

Parameter	Patient's value	Reference range
Serum sodium	115 mEq/L	135 - 145 mEq/L
Serum potassium	3.5 mEq/L	3.5 - 5.0 mEq/L
Serum chloride	98 mEq/L	96 - 106 mEq/L
Serum bicarbonate	22 mEq/L	22 - 28 mEq/L
Serum osmolality	233 mOsm/kg	275 - 295 mOsm/kg
Urine osmolality	219 mOsm/kg	300 - 900 mOsm/kg
Urine sodium	<20 mEq/L	>20 mEq/L
Blood urea nitrogen	18 mg/dL	7 - 20 mg/dL
Serum creatinine	0.9 mg/dL	0.6 - 1.3 mg/dL
Glucose	92 mg/dL	70 - 100 mg/dL
Calcium	9.2 mg/dL	8.5 - 10.5 mg/dL
Magnesium	1.7 mg/dL	1.5 - 2.5 mg/dL
Phosphate	3.5 mg/dL	2.5 - 4.5 mg/dL
Thyroid-stimulating hormone	1.6 mIU/L	0.5 - 4.7 mIU/L
Cortisol	14 mcg/dL	5 - 25 mcg/dL
Alanine aminotransferase	28 U/L	7 - 56 U/L
Aspartate aminotransferase	32 U/L	10 - 40 U/L
Alkaline phosphatase	88 U/L	44 - 147 U/L
Total bilirubin	0.9 mg/dL	0.1 - 1.2 mg/dL

The patient was initially treated with a 1-liter bolus of normal saline, followed by careful sodium correction through slow intravenous fluid administration to avoid osmotic demyelination syndrome. The target was to increase serum sodium by 6 to 8 mmol/L within the first 24 hours while also ensuring the increase remained limited once normalized. She was also placed on fluid restriction to avoid exacerbating potential fluid overload, which can occur during the correction of hypovolemic hyponatremia. Thiamine and folic acid supplementation were initiated to address potential nutritional deficiencies related to chronic alcohol use.

**Figure 1:**
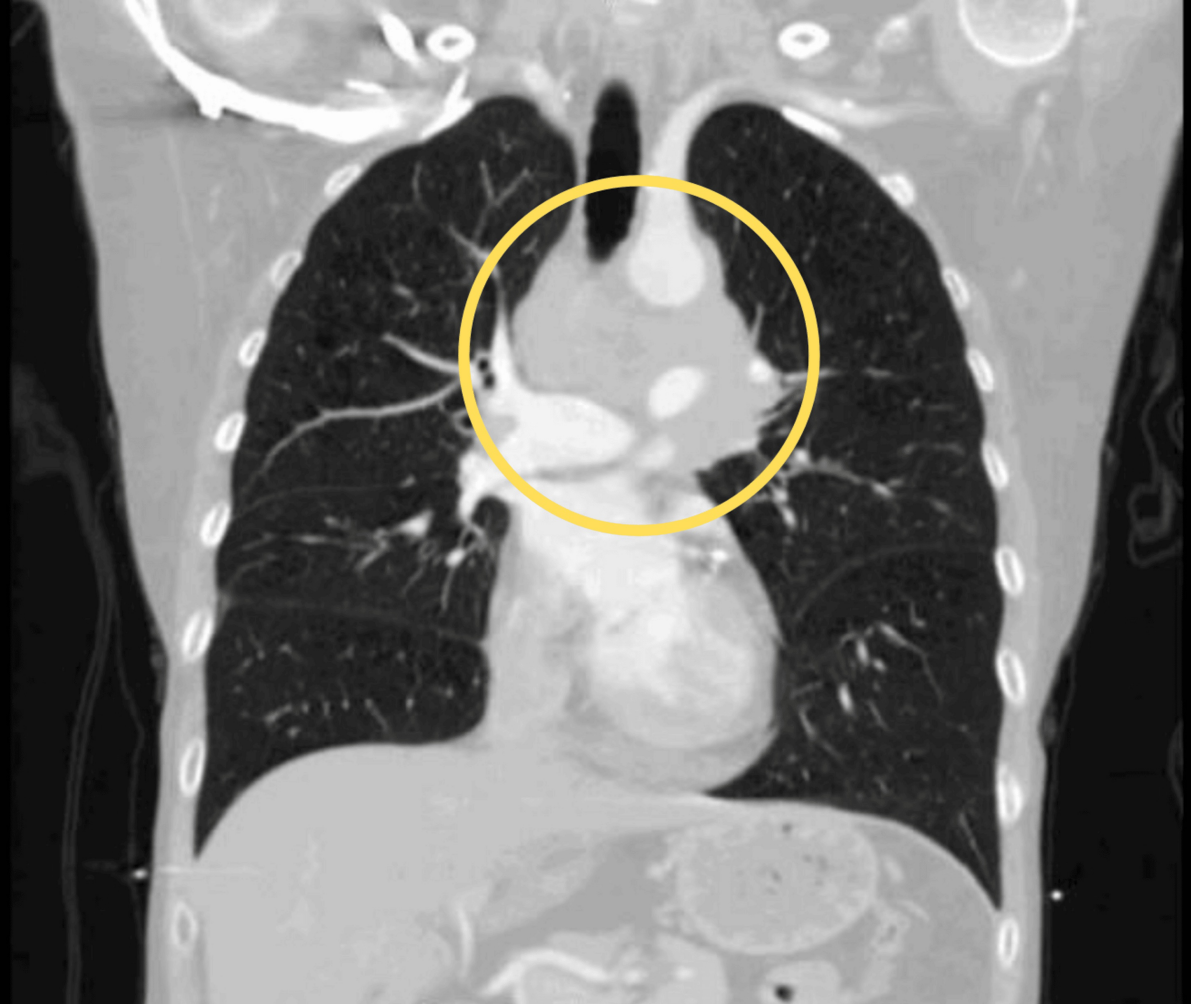
Coronal CT scan showing a precarinal mass (encircled in yellow) accompanied by multiple enlarged lymph nodes in the aortopulmonary window. CT: Computed tomography

**Figure 2:**
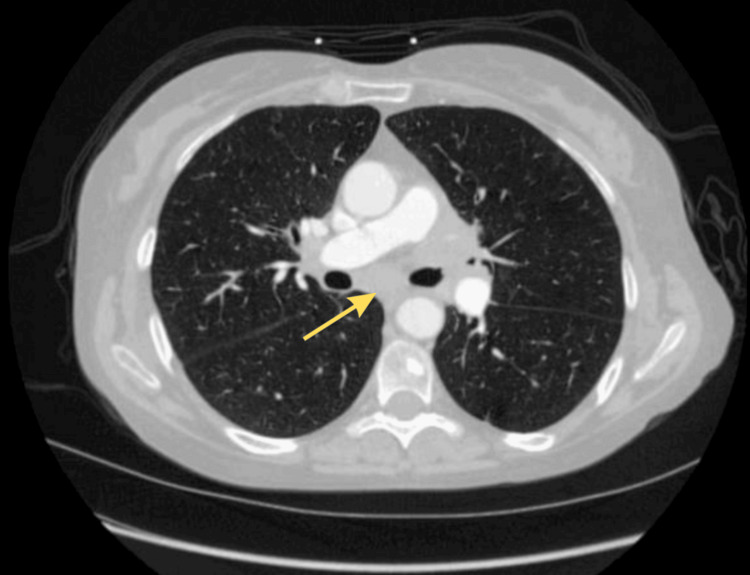
Axial CT scan showing a precarinal mass (yellow arrow) accompanied by multiple enlarged lymph nodes in the aortopulmonary window. CT: Computed tomography

**Figure 3:**
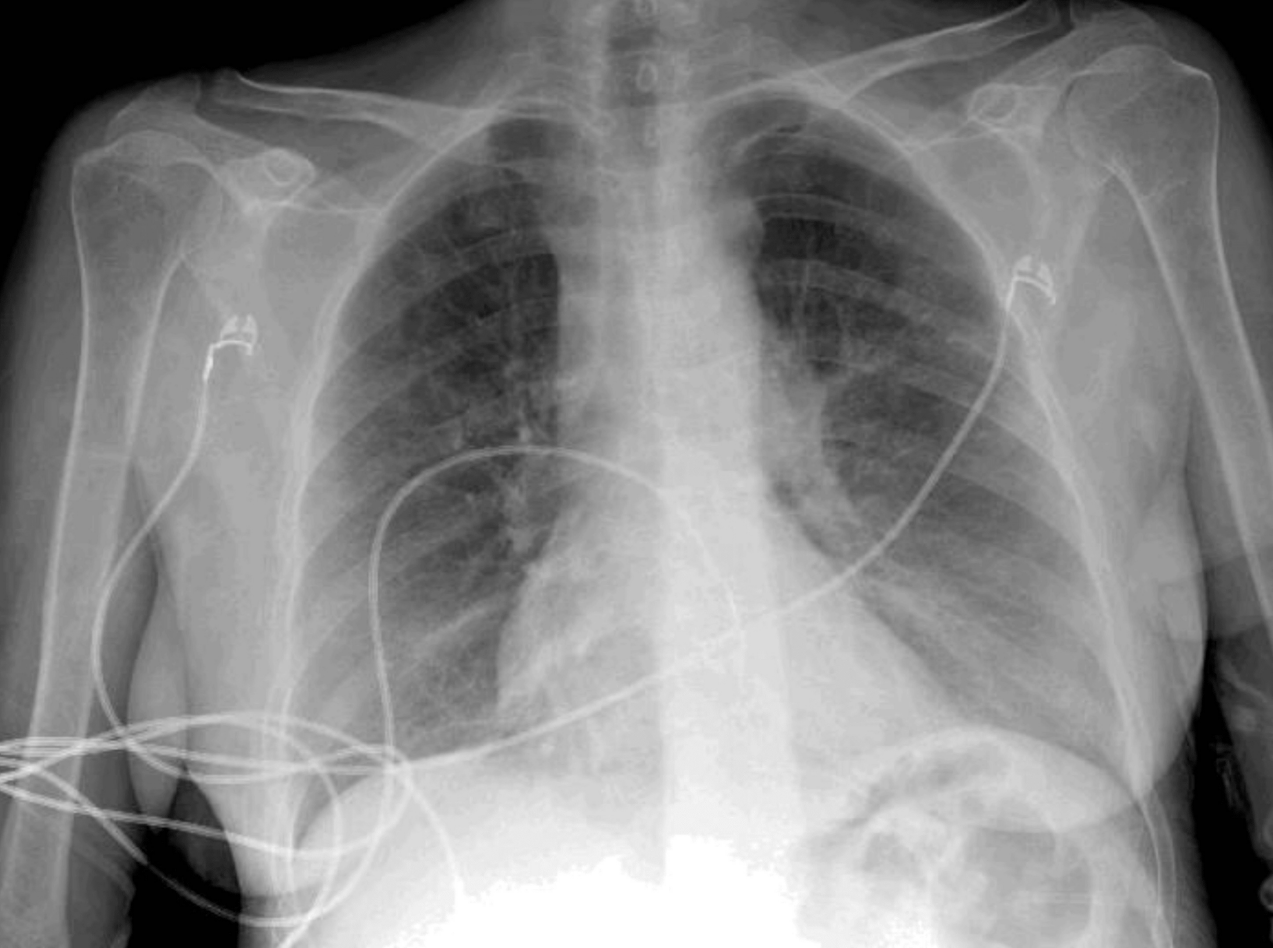
Post-bronchoscopy chest x-ray demonstrating no evidence of infiltrates or complications.

Given her significant smoking history and associated risk for malignancy, further diagnostic evaluation was pursued. A chest computed tomography (CT) scan revealed extensive mediastinal lymphadenopathy, including a 4 x 3 cm precarinal mass and multiple enlarged lymph nodes in the aortopulmonary window, raising concerns for an underlying malignancy (Figures 1, 2). To further investigate the etiology of the lymphadenopathy, the patient underwent bronchoscopy with endobronchial ultrasound and fine-needle aspiration of the affected lymph nodes. Histopathologic analysis confirmed a diagnosis of SCLC. A post-bronchoscopy chest x-ray was performed to rule out any procedural complications or infiltrates, rather than as part of the staging workup for the malignancy. The chest x-ray showed no signs of infiltrates or procedural complications (Figure 3).

Despite the confirmation of SCLC, the patient’s laboratory results continued to show persistently low urine sodium levels, consistent with possible SIADH secondary to her malignancy. Sodium levels gradually improved with ongoing cautious correction through intravenous fluids and close monitoring. Over five days of hospitalization, her serum sodium levels approached normal limits without any neurological complications. Before discharge, the patient was counseled extensively on the importance of medication adherence and the risks associated with ongoing alcohol use. She was discharged in stable condition with outpatient follow-up arranged for oncology evaluation and continued management of her hyponatremia. Plans were made to initiate chemotherapy and radiation therapy for SCLC. Coordination with her primary care physician and mental health services was also established to support her in managing alcohol use disorder and psychiatric care.

## Discussion

Hyponatremia is a common electrolyte disturbance that can result from a wide range of etiologies, necessitating a thorough and multifaceted approach to diagnosis and management. In this case, the 53-year-old female patient presented with severe hyponatremia attributed to a combination of chronic alcohol use, dehydration, and possible SIADH secondary to SCLC. The complex interplay of these factors highlights the challenges clinicians face when addressing hyponatremia in patients with concurrent medical conditions.

Chronic alcohol use is a well-documented contributor to electrolyte imbalances, primarily through its impact on fluid balance and nutritional status. Chronic alcohol consumption often leads to poor dietary intake, volume depletion, and electrolyte disturbances, as seen in this patient. In particular, the condition known as “beer potomania” occurs due to the excessive intake of beer, which is low in sodium and high in water content, resulting in dilutional hyponatremia. This condition is characterized by low serum sodium and osmolality with relatively low urine osmolality, reflecting the body's inability to excrete free water [8]. Dehydration, as a consequence of poor fluid intake and chronic vomiting, likely contributed to the development of hypovolemic hyponatremia. Additionally, beer potomania, which typically presents as hypervolemic or euvolemic hyponatremia due to excessive beer intake, may have compounded the electrolyte imbalance in this patient. Her non-adherence to medications, including hydrochlorothiazide, may have further exacerbated the electrolyte imbalance [9]. Sudden discontinuation, if it occurred, could potentially alter fluid balance in susceptible individuals.

While there is a well-established link between SCLC and SIADH, the patient's lab results do not fully align with the typical presentation of SIADH. In SIADH, we would expect to see low serum sodium, low serum osmolality, and relatively high urine osmolality (typically above 100 mOsm/kg), along with elevated urine sodium (>20 mEq/L). However, in this case, the patient's low urine sodium and relatively low urine osmolality suggest that the hyponatremia is more likely due to dehydration and poor fluid intake, rather than SIADH alone. This suggests the presence of mixed etiologies contributing to the patient's hyponatremia, with possible dehydration from alcohol use and vomiting as the primary factors. The discovery of extensive mediastinal lymphadenopathy and a precarinal mass on chest CT in this patient emphasizes the importance of comprehensive diagnostic imaging in patients with significant risk factors for malignancy. Early identification of SCLC and its associated paraneoplastic syndromes is crucial for prompt initiation of oncologic treatment and mitigation of electrolyte disturbances.

The management of hyponatremia must be individualized and guided by the underlying etiology and the patient's clinical status. In this case, initial treatment with intravenous saline was appropriate to address the patient's hypovolemic state [4]. However, careful and gradual correction of serum sodium levels was essential to avoid osmotic demyelination syndrome, a potentially devastating neurologic complication associated with rapid sodium correction. Guidelines recommend increasing serum sodium by no more than 6 to 8 mmol/L in the first 24 hours in chronic hyponatremia cases [10]. The patient's sodium levels were closely monitored, and gradual correction was achieved without complications.

This case also emphasizes the importance of a holistic approach to patient care that extends beyond immediate medical management. Addressing underlying lifestyle factors, such as alcohol use disorder, is critical for preventing recurrence and ensuring long-term health. Multidisciplinary collaboration, involving oncologists, nutritionists, addiction specialists, and primary care providers, is essential to manage the full spectrum of the patient’s needs. Supporting the patient through cancer treatment, nutritional rehabilitation, and substance use recovery requires a coordinated effort to improve outcomes and quality of life. Ultimately, this case serves as a reminder that persistent or severe electrolyte abnormalities should prompt a thorough evaluation for underlying malignancy and other systemic conditions, particularly in patients with significant risk factors.

## Conclusions

This case highlights the importance of a comprehensive evaluation for hyponatremia, particularly in patients with a history of chronic alcohol use and significant smoking. The identification of SCLC in this patient emphasizes the potential for serious underlying conditions, such as malignancy, to contribute to electrolyte imbalances. Clinicians must remain vigilant in considering malignancy as a possible cause of hyponatremia, as timely recognition and intervention can significantly improve patient outcomes. Additionally, the case highlights the importance of CT imaging, even in the absence of respiratory symptoms, to identify underlying malignancy. Given the patient’s ongoing treatment plan, ongoing monitoring of sodium levels and tumor response during chemotherapy are critical to ensure safe management and optimize outcomes. This case serves as a reminder of the complexities involved in managing patients with multiple coexisting health issues and the need for a holistic, multidisciplinary approach to care.
